# sFlt-1 Levels as a Predicting Tool in Placental Dysfunction Complications in Multiple Pregnancies

**DOI:** 10.3390/biomedicines11112917

**Published:** 2023-10-28

**Authors:** Valentina Giardini, Leonora Grilli, Alessandra Terzaghi, Lyudmyla Todyrenchuk, Caterina Zavettieri, Giulia Mazzoni, Sabrina Cozzolino, Marco Casati, Patrizia Vergani, Anna Locatelli

**Affiliations:** 1 Department of Obstetrics and Gynecology, IRCCS San Gerardo dei Tintori Foundation, University of Milano-Bicocca, 20900 Monza, Italy; grillileonora@gmail.com (L.G.); a.terzaghi1@campus.unimib.it (A.T.); c.zavettieri@campus.unimib.it (C.Z.); anna.locatelli@unimib.it (A.L.); 2 Laboratory Medicine, IRCCS San Gerardo dei Tintori Foundation, University of Milano-Bicocca, 20900 Monza, Italy

**Keywords:** angiogenic markers, sFlt-1, endothelial dysfunction, multiple pregnancies, obstetric complications, preeclampsia, hypertensive disorders in pregnancy, fetal growth restriction

## Abstract

Background: several studies have demonstrated that angiogenic markers can improve the clinical management of hypertensive disorders (HDs) and fetal growth restriction (FGR) in singleton pregnancies, but few studies have evaluated the performance of these tests in multiple pregnancies. Our aim was to investigate the role of soluble fms-like tyrosine kinase 1 (sFlt-1) in predicting adverse obstetric outcomes in hospitalized multiple pregnancies with HD (preeclampsia/gestational hypertension/uncontrolled chronic hypertension) and/or FGR in one or more fetuses. Methods: A retrospective analysis of multiple pregnancies with HD/FGR occurring after the 20th gestational week. Pregnant women were divided into two groups: women with high levels of sFlt-1 and those with low levels of sFlt-1. A value of sFlt-1 greater than or equal to 15,802 pg/mL was considered arbitrarily high, as it is equivalent to two times the 90th percentile expected in an uncomplicated full-term singleton pregnancy based on data from a prospective multicenter study (7901 pg/mL). Results: The cohort included 39 multiple pregnancies. There were no cases of birth <34 weeks, HELLP syndrome, ICU admission, and urgent cesarean sections for HD/FGR complications reported among women with low levels of sFlt-1. Conclusions: A cut-off value of sFlt-1 ≥ 15,802 pg/mL could represent a valuable tool for predicting adverse obstetric outcomes in multiple pregnancies hospitalized for HD/FGR disorders, regardless of gestational age and chorionicity.

## 1. Introduction

Multiple gestation births account for approximately 3–4% of all births. In recent decades, their incidence in industrialized countries has increased, due to advanced maternal age at time of conception and above all, due to a greater use of medically assisted procreation (MAP) [1]. These pregnancies are significantly associated with serious maternal morbidity and worse perinatal outcomes compared to singleton gestations [2,3]. Of note, women with multiple pregnancies have a higher risk of developing hypertensive disorders (HDs), such as preeclampsia (preE) or gestational hypertension, which tend to occur at an earlier gestational age and appear more severe/atypical [4]. Indeed, Laine et al. published a large study including a cohort of 16,174 twin pregnancies, in which the risk of preE in twins was found to be almost four times higher than in singletons, even after adjusting for other risk factors [5]. In addition, approximately 25–47% of twin pregnancies are affected by fetal growth restriction (FGR), depending on the definition of growth restriction [6]. FGR’s etiology is multifactorial, but its main cause is placental insufficiency [7]. Compared to age-matched singletons, twins with FGR have higher perinatal mortality and morbidity rates [8]. The diagnosis and management of HDs and FGR remains a challenge, especially regarding multiple pregnancies [9]. Several studies have demonstrated that angiogenic markers can improve the clinical management of these disorders in singleton pregnancies, but few studies have evaluated their performance in multiple pregnancies [10].

**Figure 1:**
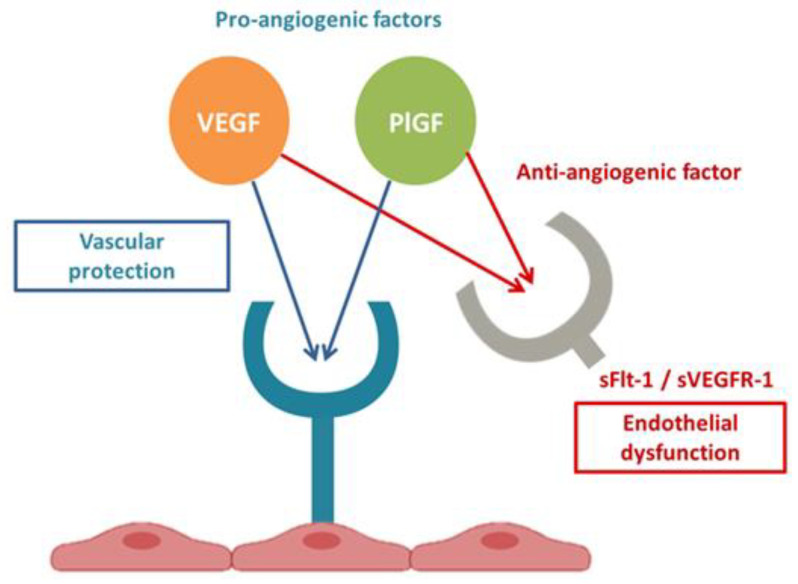
Angiogenic factor interactions. Image taken from our article about similarities between the pathogenesis of preE and COVID-19 [23].

The clinically used angiogenic markers are PlGF (Placental Growth Factor—a pro-angiogenic factor) and its decoy receptor sFlt-1 (soluble fms-like tyrosine kinase 1—an antiangiogenic factor). In normal pregnancies, there is a gradual decrease in PlGF and an increase in sFlt-1 towards the end of pregnancy. However, women with placental dysfunction, such as preE and/or FGR, have lower levels of PlGF and higher levels of sFlt-1 relative to gestational age [11]. sFlt-1 antagonizes the vascular endothelial growth factor (VEGF) and PlGF in the circulation by binding to them, preventing interaction with their endothelial receptors, creating endothelial dysfunction [12] (see Figure 1). Elevated levels of sFlt-1 in maternal serum are associated with the development of clinical signs and symptoms of preE [13] and are directly proportional to the severity of preE [14]. Placental ischemia could mediate increased sFlt-1 production [15]. Moreover, high levels of sFlt-1 have also been detected in other conditions characterized by endothelial dysfunction, such as sepsis, heart disease, acute pancreatitis, and even COVID-19, resulting in a poor prognosis [16,17,18,19,20,21,22].

Currently, the sFlt-1/PlGF ratio is considered a clinical biomarker for the early detection and prognosis of preE [24] but also a potential indicator in predicting FGR [25]. In singleton pregnancies, a sFlt-1/PlGF ratio of 38 or less can be used to exclude a diagnosis of preE in women in whom the syndrome is clinically suspected [26], while gestational age-specific sFlt-1/PlGF ratio cut-offs of >85 (20 + 0 to 33 + 6 weeks) and >110 (34 + 0 weeks to delivery) have been shown to be highly suggestive of preE [27]. In multiple pregnancies, there is no clarity on which cut-offs to use [28]. Furthermore, the available data in the literature are still focused exclusively on the sFlt-1/PIGF ratio. Since sFlt-1 is an antiangiogenic factor, toxic when in excess, our aim was to investigate its role alone in predicting an adverse obstetric outcome in hospitalized multiple pregnancies with placental dysfunction, HD, and/or FGR of one or more fetuses, regardless of chorionicity and gestational age. Specifically, we analyzed the whole sample for prepartum sFlt-1 levels using 15,802 pg/mL as the cut-off to define it as “high”, equivalent to two times the expected 90th percentile in an uncomplicated singleton pregnancy at term (≥37 weeks) [29].

## 2. Materials and Methods

A retrospective analysis of multiple pregnancies with HD and/or FGR of one or more fetuses requesting hospitalization after the 20th gestational week was performed at the Obstetrics and Gynecology Unit of IRCCS San Gerardo dei Tintori Foundation in Italy from May 2018 to December 2022.

All patients signed informed consent forms for the collection of biological materials.

Multiple pregnancies with malformed growth-restricted fetuses, intrauterine deaths, and cases of feto-fetal transfusion were excluded from the study.

Blood samples for sFlt-1 dosage were collected during hospitalization and sFlt-1 was measured on the Cobas e601 platform (Roche Diagnostics Italy), using the electrochemiluminescence immunoassay principle (REF 05109523190 and 05144671190, respectively).

In cases of multiple sFlt-1 doses during the same hospitalization stay or during more hospitalizations, the last dose before delivery was considered.

A sFlt-1 level greater than or equal to 15,802 pg/mL was arbitrarily considered high, as it is equivalent to two times the 90th percentile expected in an uncomplicated full-term (≥37 weeks) singleton pregnancy (7901 pg/mL) [29].

Pregnant women were divided into two groups: women with high levels of sFlt-1 and those with low levels of sFlt-1.

Choices relating to delivery were made independently of the value of biochemical markers, and based only on maternal-fetal clinical and laboratory parameters as reported in the guidelines.

The following data about anamnestic details were collected from medical records: type of multiple pregnancy, age, parity, pregestational obesity (body mass index—BMI ≥ 30), pre-gestational and gestational diabetes, chronic hypertension, pregnancy complications at the time of blood testing, such as HD (preE or gestational hypertension or uncontrolled chronic hypertension), FGR in at least one twin, or both (HD + FGR).

The following obstetric outcomes were recorded: gestational age at delivery, premature birth (<34 weeks), urgent caesarean section for HD/FGR problems (i.e., severe preE), HELLP (hemolysis, elevated liver enzymes, and low platelets) syndrome, maternal intensive care unit (ICU) admission for HD complications, antihypertensive therapy before delivery and at postpartum discharge.

The gestational age at the time of blood testing and the latency time between blood tests and delivery were also recorded.

PreE was defined by the most recent clinical criteria from ACOG [30]. The diagnosis of FGR was made when the abdominal circumference (AC) or estimated fetal weight (EFW) of one twin was <10th percentile and the umbilical artery pulsatility index (UA-PI) of the smaller twin >95th percentile or in cases of deflection/arrest of fetal growth with AC/EFW <10th percentile.

Discrete variables were reported as numbers and percentages, and continuous variables were reported as means and standard deviations. Differences between groups were evaluated using the Wilcoxon-Mann-Withney or Fisher’s Exact test and *p* < 0.05 was considered statistically significant.

## 3. Results

The study population included 39 multiple pregnancies: 37 twins (34 dichorionic–diamniotic DD, 2 monochorionic–diamniotic MD, 1 monochorionic–monoamniotic MM) and 2 triplets (dichorionic—triamniotic). 16 pregnancies (41%) were conceived through MAP procedures. In particular, 5 from heterologous MAP, 4 from egg donation, 1 from embryo donation, and 1 resulted from homologues MAP after cytoplasmic transfer from a donor. One pregnancy occurred after pharmacological ovulation induction.

The mean maternal age was 35 ± 6 years.

Of these 39 patients, 9 (23%) were hospitalized twice during their pregnancies; one patient being admitted 3 times.

55 sFlt-1 tests were performed, with 12 patients dosed at sFlt-1 twice—2 patients tested 3 times. The last value before delivery was considered for the analysis.

15 (38%) women had high levels of sFlt-1 (ranging 16,585 to 26,658 pg/mL) and 24 (62%) had low levels of sFlt-1 (ranging from 3504 to 15,391 pg/mL). The mean sFlt-1 was 21,654 ± 3161 pg/mL in the high levels group and 9326 ± 3163 pg/mL in the low levels group. The data are reported in Table 1.

There were no significant differences between the two groups with respect to the types of multiple pregnancies, maternal anamnestic characteristics, pregnancy complications at hospitalization, and the gestational age during the blood test at the last sFlt-1 dosage.

The latency time between blood testing and delivery was shorter in the group with high sFlt-1 levels than in the group with low sFlt-1 levels (3 ± 3 vs. 7 ± 4 days, *p* = 0.007). Delivery occurred earlier in the first group compared to the second, although the mean gestational age at delivery did not reach a significant difference between the two groups: 34 + 2 weeks in women with high levels of sFlt-1 versus 36 + 4 weeks in women with low sFlt-1 levels (*p* = 0.060).

No cases of birth 34 weeks, urgent cesarean section for HD/FGR complications, HELLP syndrome, or ICU admission were reported among women with low levels of sFlt-1, with a statistically significant difference for the first two outcomes (27% vs. 0%, *p* = 0.016 for both). It is important to underline that patients with these adverse outcomes all had sFlt-1 levels > 20,000 pg/mL.

Furthermore, women with high sFlt-1 levels required more antihypertensive therapy before delivery (47% vs. 4%, *p* = 0.002), with a difference that was not statistically significant at postpartum discharge (47% vs. 17%, *p* = 0.068). In particular, among the patients with high sFlt-1 levels who required antihypertensive therapy, there were 2 women with chronic hypertension, for whom therapy was boosted during hospitalization.

## 4. Discussion

Angiogenic markers are increasingly becoming part of our clinical practice for the screening, diagnosis, and monitoring of placental dysfunction disorders, especially preE and FGR. However, many questions remain about which markers to dose, the cutoffs to use, and their role in specific obstetric conditions, including their application in the case of multiple pregnancies [28,31].

The placenta is the main source of circulating pro- and anti-angiogenic factors during pregnancy. Consequently, placental dysfunction can result in an anti-angiogenic imbalance, leading to generalized maternal endothelial injury, which underlies the clinical syndrome of preE [32].

While several studies in singleton pregnancies showed a correlation between a high sFlt-1/PIGF ratio, preE onset, and a short mean time to delivery, its use in multiple pregnancies is still a matter of debate. For singleton pregnancies, an sFlt-1/PlGF ratio of 38 or less can be used to exclude a diagnosis of preE in women clinically suspected of having the syndrome [26]. In a recent study, De La Calle et al. proposed the use of similar reference ranges for twins and singleton pregnancies until 29 weeks of gestation. From the 29th week of gestation onwards, the median, 5^th^, and 95^th^ percentile values for the sFlt-1/PlGF ratio appear to become higher in twin pregnancies. Notably, sFlt-1 values were higher in women with twin pregnancies across all gestational windows, while PlGF values were similar or higher in twin pregnancies versus singleton pregnancies [33].

Their results were in agreement with the twin study conducted by Binder et al., which defined a cutoff of lower than 38, regardless of gestational age at sampling and chorionicity, for ruling out the occurrence of delivery for preE within 1 or 2 weeks, with a negative predictive value of 98.8% or 96.4%, respectively [34]. In contrast, Saleh et al. demonstrated that this cutoff is not applicable to twin pregnancies for predicting the absence of preE or the absence of adverse pregnancy outcomes, underlining the presence of higher serum sFlt-1 levels in twins versus singleton gestations [35].

Meanwhile, other studies have tried to propose sFlt-1/PlGF reference values in twin pregnancies. Dröge et al. proposed 53 as an optimal cutoff for the sFlt-1/PlGF ratio in diagnosing preE with a sensitivity of 94.4% and a specificity of 74.2% [36]. Shinohara et al. suggested a cutoff of 22.2 when measured between 28 and 30 weeks and 6 days of gestation to predict preE onset within 4 weeks [37]. Martinez-Varea et al. reported that a ratio of ≥17 at 24 weeks among all twin pregnancies was associated with a significantly increased frequency of placental dysfunction-related diseases, including preE and FGR [38]. Indeed, pregnancies complicated by FGR are characterized by low levels of PlGF and higher median sFlt-1/PlGF ratios compared to gestational-age-matched pregnancies, but the role of sFlt-1/PIGF as a screening tool for predicting FGR in singletons is currently a subject of intense debate, and there is no clarity on which cutoff values to use [31,39].

Furthermore, Rana et al. demonstrated that a high sFlt-1/PlGF ratio was associated with adverse maternal and perinatal outcomes, even in twin pregnancies with suspected preE [40]. In contrast, Karge et al. did not find a predictive value of the sFlt-1/PIGF ratio for adverse perinatal outcomes, but they confirmed that the mean time to delivery was inversely related to the sFlt-1/PIGF ratio, similar to singleton pregnancies and speculated that the sFlt-1/PIGF ratio could predict selective FGR (s-FGR) [41].

In conclusion, the available data in the literature still need to be defined and are still contradictory and scarce. In addition, more recently published studies have exclusively focused on establishing a reference cutoff for the sFlt-1/PIGF ratio without considering the possible role of individual markers, especially sFlt-1.

sFlt-1 is the commercially detectable endogenous anti-angiogenic protein. Both in vitro and in vivo models suggest that placental ischemia/hypoxia increases the expression of sFlt-1 [42,43]. Excess placental production of sFlt1 contributes to hypertension and proteinuria noted in patients with preE [44], and are directly proportional to the severity of preE [14]. Jantsch et al. reported that multiple pregnancies have an increased risk of oxidative stress and reduced antioxidant capacity [45]. Indeed, several studies have reported higher concentrations of sFlt-1 in multiple pregnancies compared to singletons [46]. These findings may justify the increased risk of preE in twin pregnancies [5]. Increased circulating sFlt1 is associated with decreased circulating levels of free VEGF and PlGF [44]. For this reason, the sFlt1/PlGF ratio has shown better diagnostic performance than single biomarkers, especially for predicting preE in singleton pregnancies [47].

Regarding multiple pregnancies, Kozłowski et al. showed that levels of angiogenic biomarkers differ between dichorionic and monochorionic twins, with a significantly higher concentration of sFlt-1 in dichorionic in comparison to monochorionic pregnancies in both the first and third trimesters, suggesting a possible relationship with the higher incidence of preE observed in dichorionic twin gestations [48]. Furthermore, sFlt-1 is a strong predictor of adverse outcomes, not only in obstetrics [16,17,18,19,20,21,22].

In light of these considerations, we think that there is a threshold limit of tolerance by the mother to this anti-angiogenic factor; excessive sFlt-1 levels are toxic. Therefore, we tested the hypothesis that sFlt-1 alone could be a better marker of adverse outcome in multiple pregnancies, regardless of chorionicity and gestational age.

In our analysis, we used an arbitrary toxic cutoff value for sFlt-1, greater than or equal to 15,802 pg/mL, equivalent to two times the 90th percentile expected in an uncomplicated full-term (≥37 weeks) singleton pregnancy (7901 pg/mL) [29].

In our series, no cases of birth <34 weeks, urgent cesarean section for HD/FGR complications, HELLP syndrome, or ICU admission were reported among women with low levels of sFlt-1. This difference was statistically significant for the first two outcomes. Notably, the patients with these obstetrics complications all had sFlt-1 > 20,000 pg/mL. These results confirm the importance of monitoring and focusing on sFlt-1 levels for the assessment of adverse obstetric outcomes, particularly maternal ones.

Rana et al. demonstrated that a high sFlt-1/PlGF ratio was associated with adverse maternal and perinatal outcomes in twin pregnancies with suspected preE. They compared 52 women (65.8%) who experienced an adverse outcome with 27 women (34.2%) without an adverse outcome. They found a higher median sFlt-1 in the first group, but with lower sFlt-1 values than our cutoff: 11,461.5 pg/mL (8794.0–14,847.5) versus 7495.0 pg/mL (3498.0–10,482.0; *p* = 0.0004) [40].

With respect to the use of the sFlt-1/PlGF ratio, when single angiogenic marker assays are involved, standardization between the assay methods is required to allow for adequate comparison between studies. Our assays of sFlt-1, for example, are not comparable with those of the study by Maynard et al., who used another type of commercial kit (R&D Systems, Minneapolis, MN, USA) [46]. However, they showed that maternal serum sFlt-1 levels and the sFlt-1 to PlGF ratio are higher in women with twins or triplets compared to high-risk singleton pregnancies in the late second and third trimesters.

In addition, our data confirm a correlation between high levels of sFlt-1 and the timing of delivery. The latency time between blood testing and delivery was shorter in this group, suggesting that sFlt-1 could also be a potential tool to identify women at risk for imminent delivery.

Our study has several limitations, including its retrospective and monocentric nature and small sample size. Prospective data with larger numbers and standardization between assay methods are needed to define which cutoffs of sFlt-1 are indicative of maternal-fetal complications that require careful obstetrical surveillance and a definite timing of delivery.

## 5. Conclusions

There is an urgent need for standardized cutoffs of angiogenic markers to be applied in obstetric clinical practice. Our study focuses on the importance of defining a “toxic cutoff” for sFlt-1, because excessive sFlt-1 levels are associated with serious obstetric complications. Multiple pregnancies are at a higher risk for many adverse effects, including an increased level of oxidative stress, and this risk is proportional to the number of fetuses. sFlt-1 is released from the trophoblast in response to various stressors, including oxidative stress. These considerations may explain why sFlt-1 could be used to assess the degree of placental dysfunction, and multiple pregnancies constitute a model for better defining pregnancy tolerance to sFlt-1.

Based on the analysis of our cases, a sFlt-1 cutoff value of ≥15,802 pg/mL could represent a valuable tool for predicting adverse obstetric outcomes in multiple pregnancies hospitalized for an HD/FGR disorder, regardless of gestational age and chorionicity.

## Figures and Tables

**Table 1:** Anamnestic details and obstetric outcomes comparing women with high levels of sFlt-1 versus low levels of sFlt-1 [Mean; standard deviation—SD; n; (%)].

Multiple Pregnancies—n = 39	High Levels of sFlt-1	Low Levels of sFlt-1	*p* Value
	sFlt-1 ≥ 15,802 pg/mL	sFlt-1 < 15,802 pg/mL	
	n = 15 (38)	n = 24 (62)	
**Types**			
DD twins	12 (80)	22 (92)	0.354
MD twins	1 (7)	1 (4)	1.000
MM twins	1 (7)	0	0.384
Triplets	1 (7)	1 (4)	1.000
**Maternal anamnestic characteristics**			
Age (years)	37 ± 5	35 ± 6	0.136
Nulliparous	11 (73)	19 (79)	0.711
Obesity (BMI > 30 kg/m^2^)	3 (20)	5 (21)	1.000
Diabetes/GDM	4 (27)	4 (17)	0.685
Chronic hypertension	2 (13)	0	0.141
**sFlt-1**			
GA at blood test (weeks.days)	33.6 ± 3.5	35.4 ± 1.3	0.136
Latency time between blood test and delivery (days)	3 ± 3	7 ± 4	**0.007**
**Pregnancy complications at blood test**			
HD	9 (60)	9 (38)	0.202
HD + FGR	3 (20)	6 (25)	1.000
FGR	3 (20)	9 (38)	0.305
**GA at 1st hospitalization for HD/FGR (weeks.days)**	32.5 ± 4.2	34.6 ± 1.3	0.126
**GA at delivery (weeks.days)**	34.2 ± 3.5	36.4 ± 1.0	0.060
**Adverse outcomes**			
Birth < 34 weeks	4 (27)	0	**0.016**
Urgent cesarean section for HD/FGR	4 (27)	0	**0.016**
HELLP syndrome	2 (13)	0	0.141
ICU admission for HD complications	2 (13)	0	0.141
Antihypertensive therapy before delivery	7 (47)	1 (4)	**0.002**
Antihypertensive therapy at postpartum discharge	7 (47)	4 (17)	0.068

Legend: DD twins—dichorionic–diamniotic twins, MD twins—monochorionic–diamniotic twins, MM twins—monochorionic–monoamniotic twins, BMI—body mass index, GDM—gestational diabetes mellitus, GA—gestational age, HD—hypertensive disorders: preE, gestational hypertension, uncontrolled chronic hypertension, FGR—fetal growth restriction, HELLP syndrome—hemolysis, elevated liver enzymes, and low platelets syndrome, ICU—intensive care unit.

## Data Availability

The data that support the findings of this study are available on request from the corresponding author.
